# Supplementary material for: Higher-order interactions in neuronal function: From genes to ionic currents in biophysical models
Source: Proc Natl Acad Sci U S A. 2025 Sep 29;122(40):e2500048122. doi: 10.1073/pnas.2500048122 (PMC12519081; doi:10.1073/pnas.2500048122)
Supplement: Supplementary file 1 — Appendix 01 (PDF) [file pnas.2500048122.sapp.pdf]

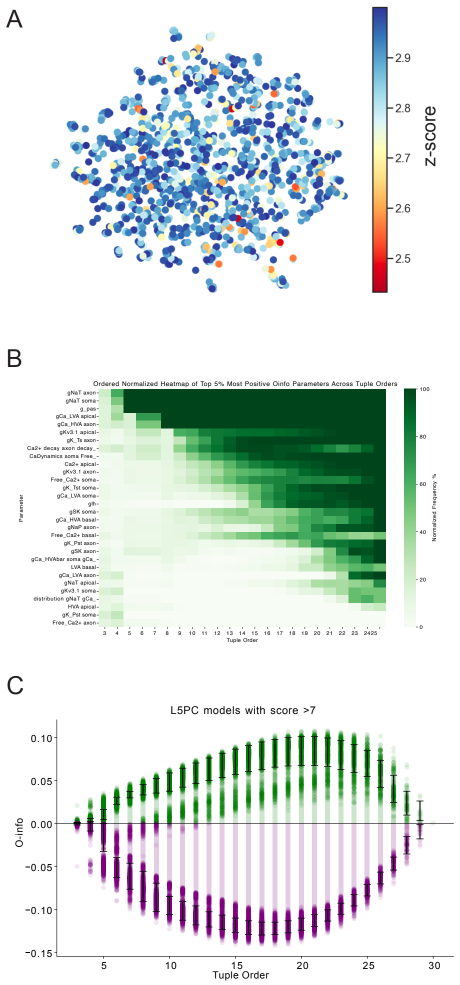

Figure S1: A. UMAP representation of model parameters. The colors indicate the fitness of the model, quantified as the z-score for the corresponding parameter set. B. Normalized frequency map highlighting parameters that frequently occur in high-redundancy tuples. C. O-information plot showing the relationship between tuple number (number of parameters) and O-information values for models L5PC models that have score value larger than 7. Each dot represents the O-information value for a specific tuple. The bars and whiskers illustrate the mean and standard deviation of O-information values at each tuple order. The color encodes positive (green) and negative (purple) O-info values.

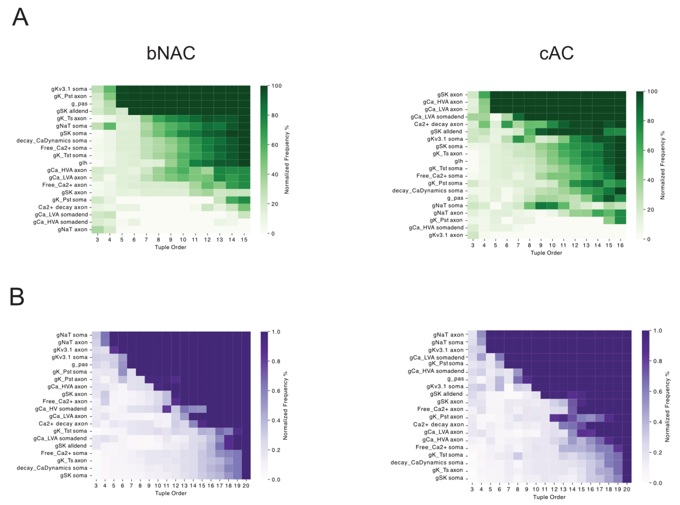

Figure S2: A. Normalized frequency map showing parameters that frequently appear in high-redundancy tuples based on Oinfo calculations for cAC and bNAC neuronal models. B. Normalized frequency map for parameters in high-synergy tuples, similar to panel.

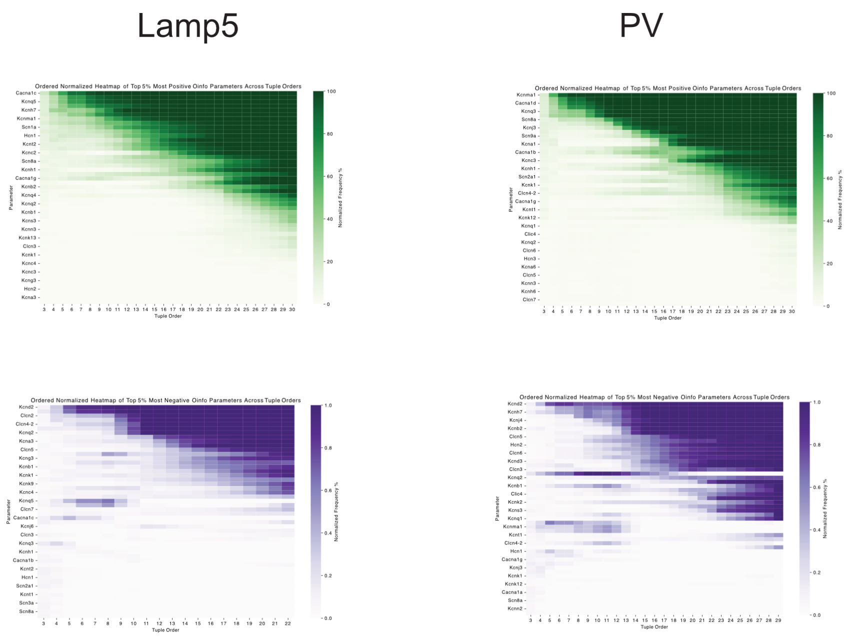

Figure S3: Normalized frequency maps of parameters frequently appearing in high-redundancy (top) and high-synergy (bottom) tuples for Lamp5 and Pvalb neurons.

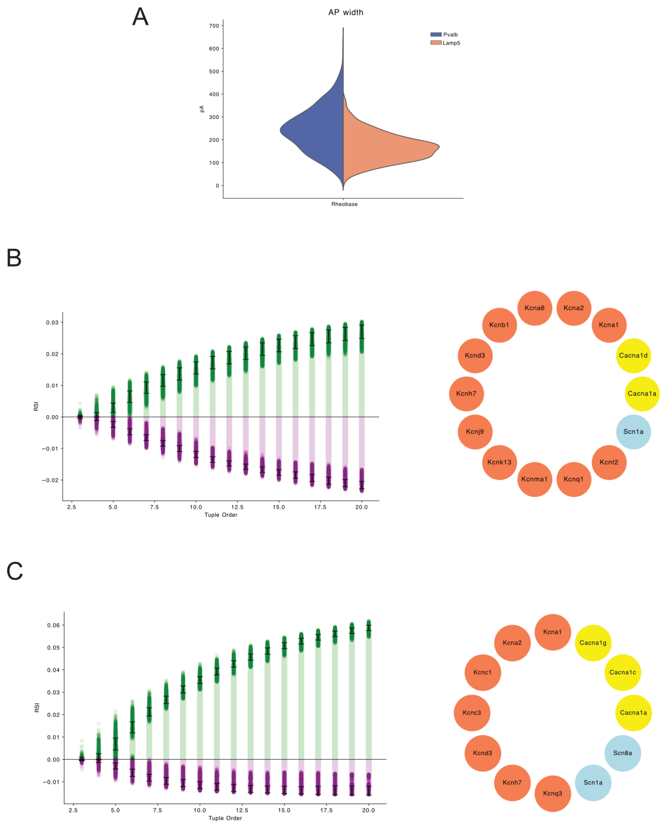

Figure S4: A. Distribution of mean frequencies (Hz) for cells from Lamp5 and Pvalb neuronal types. B. RSI values plotted against tuple numbers (right), along with the set of genes that yield the highest RSI value (left) for Lamp5 neurons. C. Similar RSI values and gene sets as in panel B, but for Pvalb neurons.

PV (AP Width)

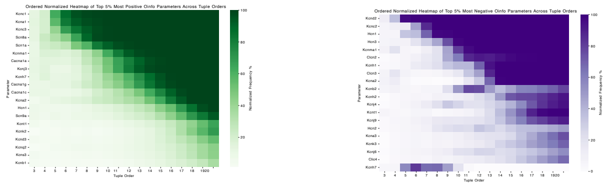

PV (Rheobase)

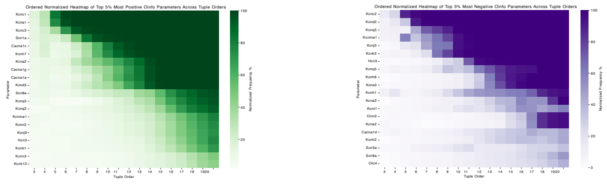

Lamp5 (AP Width)

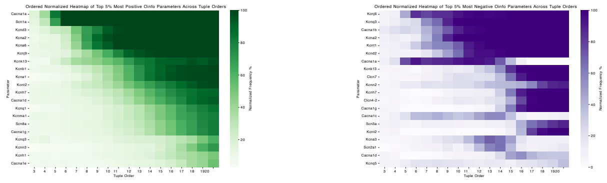

Lamp5 (Rheobase)

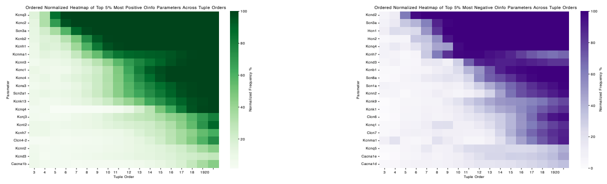

Figure S5: Normalized frequency maps of parameters frequently appearing in high-redundancy (left) and high-synergy (right) tuples. Data correspond to gene expression, action potential width (AP width), and mean frequency features for Lamp5 and Pvalb neurons.

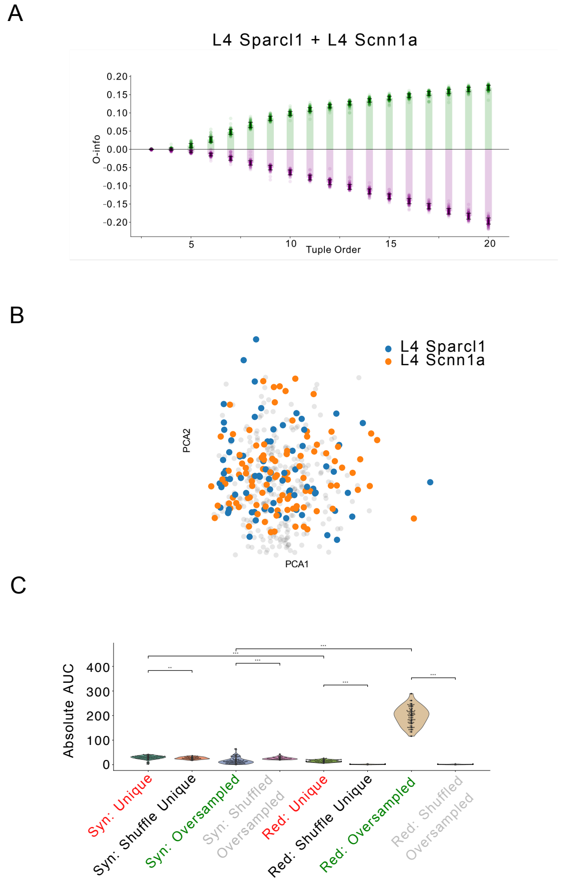

Figure S6: **A.** O-info decomposition restricted to models from the L4 Sparcl1+ and L4 Scnn1a+ excitatory neuron subtypes. Green and purple curves represent the mean ± SD of positive and negative O-information values, respectively, as a function of tuple order. **B.** Projection of the parameter sets corresponding to L4 Sparcl1+ and L4 Scnn1a+ gene expression onto the first two principal components. Colors indicate subtype identity. **C.** Distribution of absolute O-info AUC values for both synergy (minima) and redundancy (maxima) across the Unique, Oversampled, and Shuffled groups. Violin plots display group-wise variability, with statistical comparisons performed using Mann-Whitney U tests ($p < 0.001$).

SI Methods

Detailed biophysical models

Detailed biophysical model was constructed based on the patch clamp electrophysiological recordings of L5PC, cAC and bNAC cells as previously described and available online [1]. In the experimental dataset [1, 2], each recorded cell was probed with a series of stimulation protocols: (1) IDrest, consisting of depolarizing steps with a sampling frequency of 10 kHz and duration of 2 seconds; (2) IDthresh, also with depolarizing steps at a 10 kHz sampling frequency and 2-second duration; (3) APWaveform, with depolarizing steps at a higher sampling frequency of 50 kHz for 50 ms; (4) IV, a sequence of current steps ranging from hyperpolarization to depolarization, sampled at 10 kHz over 3 seconds. A holding current was applied to maintain the membrane potential at -70 mV (prior to a liquid junction potential correction of 14 mV). In total, the features extracted from the recordings include voltage measurements, such as voltage after stimulus, baseline voltage, maximum voltage relative to baseline, and voltage deflections during stimulus onset and offset. Action potential (AP) properties, including AP amplitude, specific amplitudes (AP1, AP2, and last AP), AP half-width, and after-hyperpolarization (AHP) depth. Firing characteristics, such as mean firing frequency, total spike count, burst number, and ISI coefficient of variation. Timing-related features, such as time to first and last spikes and the inverse timing of successive ISIs, further describe the temporal aspects of neuronal firing. Additionally, resistance and current measurements (input resistance, holding current, and threshold current) and the decay time constant following stimulus were assessed to characterize the cell's intrinsic properties. The full description of the feature can be found at `https://efel.readthedocs.io/en/latest/eFeatures.html`.. These features were extracted for step protocols, the intensity of the step protocols reflects the fraction of the rheobase current (current necessary to elicit a single spike). The full list of protocols and features is can be found in SI.Table 1.

The neuronal models were implemented in NEURON simulator [3]. We used a set of ionic mechanisms described previously, the table with mechanisms specific for each electrical type can be found in SI.Table 2.

Morphologies were manually reconstructed. The axons and their branches were replaced by a synthetic axon section consisting of an AIS (60 um) followed by a myelinated axon segment of 1,000 um. To define the optimization target for each cell type, we computed a feature vector representing the mean electrophysiological properties across multiple recorded neurons. As such, each biophysical model in our study is optimized to match this canonical feature set, rather than reproducing variability across biological replicates. To optimize these models, we employed an MCMC procedure as described in [4], with the cost computed as:

$$C(\mathbf{p}) = \max_i \frac{|f_i(\mathbf{p}) - f_{\exp,i}|}{\sigma(f_{\exp,i})} = \max_i z_i(\mathbf{p}), \tag{1}$$

where z_i are the absolute z-scores for each feature i and simulated feature values are represented as a vector $\mathbf{f} = (f_0, \ldots, f_k)$, and the parameters are denoted as $\mathbf{p} = (p_0, \ldots, p_n)$.

From the resulting parameter sets, we selected models with an optimization cost within 3 standard deviations for cAC and bNAC, yielding 63,408 cAC

models and 86,361 bNAC models. For L5PC, we selected models with an optimization cost within 5 standard deviations, resulting in 110,128 L5PC models.

Detection of Stabilization Points

To identify the stabilization point in the O-info (or RSI) value series, we employed a derivative-based algorithm. This approach utilizes the first and second derivatives of the RSI curve to detect the point where the data stabilizes, as follows:

- **Data Extraction:** For each tuple, the maximum RSI value is extracted, forming a series of values $y = \{y_1, y_2, \ldots, y_n\}$ corresponding to tuple indices $x = \{1, 2, \ldots, n\}$.
- **Derivative Calculation:** The first derivative $y'(x)$ is computed as the rate of change between consecutive RSI values:

  $$y'(x) = \frac{\Delta y}{\Delta x},$$

  where $\Delta y = y_{i+1} - y_i$ and $\Delta x = x_{i+1} - x_i$. Subsequently, the second derivative $y''(x)$ is calculated to capture the rate of change in the slope:

  $$y''(x) = \frac{\Delta y'(x)}{\Delta x}.$$

- **Stabilization Point Detection:** The stabilization point is identified as the index where the second derivative $y''(x)$ attains its most negative value, corresponding to the steepest concave point in the RSI curve. Mathematically:

  $$x_{\text{stab}} = \arg\min y''(x).$$

  This point marks the transition from steep decline to stabilization in the curve.

Clustering and analysis of the single cell mRNA from Patch-seq data

Data preprocessing and clustering were performed on single-cell RNA sequencing data containing expression profiles for thousands of genes across cells [5]. Ground truth cell type labels were used to evaluate clustering performance. The analyses and clustering were implemented using Python libraries, scanpy [6] was used for bio-informatics analysis (preprocessing and clustering workflows).

Gene subsets were selected as follows:

- Ion Channel Subset: A predefined list of ion channel genes was used to extract a subset of the expression matrix.
- Differentially Expressed Gene (DEG) Subset: DEGs were identified using statistical tests (e.g., t-tests) to compare expression levels across conditions.

- Random Subsets: Control subsets were generated by selecting 1,000 random gene sets, each matching the size of the ion channel subset.

Normalization was applied to ensure consistency in expression data:

- Log-transformation ($log_2(counts + 1)$) was used to normalize skewed distributions.
- Standardization was performed to scale each gene's expression to have zero mean and unit variance.

Dimensionality reduction was conducted using Principal Component Analysis (PCA) to reduce data dimensionality while retaining features explaining most of the variance (e.g., 90%).

Clustering was performed using the Leiden algorithm, which is optimized for detecting communities in graphs derived from high-dimensional data. Key steps included:

- Graph Construction: A k-nearest neighbor (kNN) graph was constructed from the principal components, with edges indicating cell similarities.
- Community Detection: The Leiden algorithm partitioned cells into clusters, with the number of clusters determined adaptively from the graph structure.
- Evaluation: Clustering results were compared to ground truth cell type labels using the Adjusted Rand Index (ARI). ARI measures label similarity, correcting for chance, with a score of 1 indicating perfect agreement.

Clustering was used to assess the specificity of ion channel, randomly selected and DEG subsets. The same preprocessing and clustering steps were repeated for 1,000 random gene subsets. ARI scores were computed for each run and compared to the ground truth labels. The resulting distribution of ARI scores served as a baseline for comparison.

Selection of the High-Covariance Model Subset

To assess how structured co-variation among model parameters influences high-order interactions, we identified a subset of models characterized by elevated inter-parameter dependencies using a principal component analysis (PCA)-based selection strategy.

Starting from the full ensemble of valid conductance-based models for cAC interneurons (i.e., models with cost ¡ 3), we performed PCA on the matrix of normalized parameters (20 per model). We retained the top N = 5 principal components, which captured 70% of variance across the parameter space. We then reconstructed each model from its projection onto these top components and quantified the residual error between the original and reconstructed parameter vectors using the L2 norm. We ranked all models by their reconstruction error and selected the top 1000 models with the lowest residuals as our high-covariance subset.

Mutual Information Computation and Significance Testing

To quantify the statistical dependency between model parameters and extracted features, we computed pairwise mutual information (MI) using a non-parametric estimator. For each parameter-feature pair, we estimated MI based on histogram-based discretization. To assess statistical significance, we applied a bootstrap procedure for each MI value. Specifically, we generated a null distribution by independently shuffling one variable in the pair (1000 permutations), and re-computing the MI to obtain an empirical p-value. These p-values were collected into a matrix and subsequently corrected for multiple comparisons using the Benjamini–Hochberg procedure (FDR control at $\alpha = 0.05$). After correction, only MI values that passed the FDR threshold and exceeded a minimal effect size threshold of 0.1 were considered significant and visualized (e.g., in the network graph and circos plots).

Bootstrap Estimation of high order measurements

To assess the robustness and variability of higher-order information metrics (O-information and Redundancy-Synergy Index, RSI), we implemented a bootstrap resampling procedure. Specifically, we drew 50 independent bootstrap samples, each consisting of 1000 (unless stated differently) for the MCMC generated models or 60% of the total sample number for the genetics data selected without replacement from the original model population for each cell type. For each resampled dataset, we computed O-information or RSI across tuple orders. This allowed us to estimate the variability of maximum and minimum values per order and to derive 95% confidence intervals from the bootstrap distributions (based on the 2.5th and 97.5th percentiles). In the figures, we plot all individual bootstrap curves along with shaded confidence envelopes, providing a quantitative assessment of the stability of synergy- or redundancy-dominated regimes. This procedure was applied uniformly across all datasets analyzed in the manuscript, including both biophysical model populations and transcriptomic data, to enable direct comparison of the robustness of inferred information-theoretic structures. However, we did not apply bootstrapping to datasets with fewer than 100 samples, or to data that includes heterogeneous clusters with small sample sizes (e.g., 10–30 samples), due to the risk of distortion from resampling limited or unevenly distributed data. In such cases, bootstrapping can over- or underrepresent minor clusters, disrupt underlying population structure, and yield unreliable estimates of high-order interactions. Given the sensitivity of O-info to subtle correlation patterns, we instead relied on direct estimation using the full available data and reported values without confidence intervals.

References

[1] Maria Reva, Christian Rössert, Alexis Arnaudon, Tanguy Damart, Darshan Mandge, Anıl Tuncel, Srikanth Ramaswamy, Henry Markram, and Werner Van Geit. A universal workflow for creation, validation, and generalization of detailed neuronal models. *Patterns*, 4(11), 2023.

[2] Henry Markram, Eilif Muller, Srikanth Ramaswamy, Michael W Reimann, Marwan Abdellah, Carlos Aguado Sanchez, Anastasia Ailamaki, Lidia Alonso-Nanclares, Nicolas Antille, Selim Arsever, et al. Reconstruction and simulation of neocortical microcircuitry. *Cell*, 163(2):456–492, 2015.

[3] Nicholas T Carnevale and Michael L Hines. *The NEURON book*. Cambridge University Press, 2006.

[4] Alexis Arnaudon, Maria Reva, Mickael Zbili, Henry Markram, Werner Van Geit, and Lida Kanari. Controlling morpho-electrophysiological variability of neurons with detailed biophysical models. *Iscience*, 26(11), 2023.

[5] Nathan W Gouwens, Staci A Sorensen, Fahimeh Baftizadeh, Agata Budzillo, Brian R Lee, Tim Jarsky, Lauren Alfiler, Katherine Baker, Eliza Barkan, Kyla Berry, et al. Integrated morphoelectric and transcriptomic classification of cortical gabaergic cells. *Cell*, 183(4):935–953, 2020.

[6] F Alexander Wolf, Philipp Angerer, and Fabian J Theis. Scanpy: large-scale single-cell gene expression data analysis. *Genome biology*, 19:1–5, 2018.

**E-type: bNAC, cNAC**
**Protocol: IDThresh/IDrest 150, 200, 250, 300** %   E-features: voltage_base, voltage_after_stim, AP_amplitude, APlast_amp, AHP_depth, inv_time_to_first_spike, time_to_last_spike, inv_first_ISI, inv_second_ISI, inv_third_ISI, inv_fourth_ISI, inv_fifth_ISI, mean_frequency
**Protocol: APWaveform 360** %   E-features: AP_amplitude, AP1_amp, AP_duration_half_width, AHP_depth
**Protocol: IV -100** %   E-features: voltage_deflection, voltage_deflection_begin
**Protocol: IV -20** % **(Rin)**   E-features: ohmic_input_resistance_vb_ssse, voltage_base
**Protocol: IV 0** % **(RMP)**   E-features: voltage_base, Spikecount
**Protocol: RinHoldCurrent**   E-features: bpo_holding_current
**Protocol: Threshold**   E-features: bpo_threshold_current
**E-type: cAC**
**Protocol: IDThresh/IDrest 140, 200, 250, 300** %   E-features: voltage_base, voltage_after_stim, AP_amplitude, APlast_amp, AHP_depth, inv_time_to_first_spike, time_to_last_spike, inv_first_ISI, inv_second_ISI, inv_third_ISI, inv_fourth_ISI, inv_fifth_ISI, mean_frequency
**Protocol: APWaveform 360** %   E-features: AP_amplitude, AP1_amp, AP_duration_half_width, AHP_depth
**Protocol: IV -100** %   E-features: voltage_deflection, voltage_deflection_begin
**Protocol: IV -20** % **(Rin)**   E-features: ohmic_input_resistance_vb_ssse, voltage_base
**Protocol: IV 0** % **(RMP)**   E-features: voltage_base, Spikecount
**Protocol: RinHoldCurrent**   E-features: bpo_holding_current
**Protocol: Threshold**   E-features: bpo_threshold_current
**E-type: cADPYR**
**Protocol: APWaveform 320** %   E-features: AP_amplitude, AP1_amp, AP2_amp, AP_duration_half_width, AHP_depth
**Protocol: IV -100** %   E-features: voltage_deflection, voltage_deflection_begin
**Protocol: IDrest & IDthresh 150, 200, 280** %   E-features: voltage_base, voltage_after_stim, AP_amplitude, APlast_amp, AHP_depth, inv_time_to_first_spike, time_to_last_spike, inv_first_ISI, inv_second_ISI, inv_third_ISI, inv_fourth_ISI, inv_fifth_ISI, mean_frequency
**Protocol: SpikeRec 600** %   E-features: decay_time_constant_after_stim, voltage_after_stim, Spikecount
**Protocol: IV -20** % **(Rin)**   E-features: ohmic_input_resistance_vb_ssse, voltage_base
**Protocol: IV 0** % **(RMP)**   E-features: voltage_base, Spikecount
**Protocol: RinHoldCurrent**   E-features: bpo_holding_current
**Protocol: Threshold**   E-features: bpo_threshold_current

Table 1: Relevant protocols and e-features for bNAC, cAC, and cADPYR e-types in a single column format.

**E-type: cADPYR**
**Soma**<br>CaDynamics, HVA Ca, LVA Ca, Kv3.1, Ca-activated K, Persistent K, Transient K, Transient Na<br>**AIS**<br>CaDynamics, HVA Ca, LVA Ca, Kv3.1, Ca-activated K, Persistent K, Transient K, Transient Na, Persistent Na<br>**Dendrites**<br>CaDynamics, HVA Ca, LVA Ca, Kv3.1 (Apical), Transient Na (Apical; Decaying), HCN (Apical and Basal; Exponential increasing towards terminals)
**E-types: bNAC, cAC**
**Soma**<br>CaDynamics, HVA Ca, LVA Ca, Ca-activated K, Transient Na, Kv3.1, Persistent K, Transient K, HCN<br>**AIS**<br>CaDynamics, HVA Ca, LVA Ca, Ca-activated K, Transient Na, Kv3.1, Persistent K, Transient K<br>**Dendrites**<br>CaDynamics, HVA Ca, LVA Ca, Ca-activated K, HCN (Exponential increasing towards terminals)

Table 2: Active parameters and their compartmental placement for cAC, bNAC, and cADPYR e-types.
